# Safety and efficacy of a 100 % dimethicone pediculocide in school-age children

**DOI:** 10.1186/s12887-015-0381-0

**Published:** 2015-06-20

**Authors:** Erin Speiser Ihde, Jeffrey R. Boscamp, Ji Meng Loh, Lawrence Rosen

**Affiliations:** The Deirdre Imus Environmental Health Center®, Hackensack University Medical Center, 30 Prospect Ave, Hackensack, NJ 07601 USA; Hackensack University Medical Center, The Joseph M. Sanzari Children’s Hospital, 30 Prospect Avenue, Hackensack, NJ 07601 USA; Dept. of Mathematical Sciences, NJ Institute of Technology - University Heights, Newark, NJ 07102 USA

**Keywords:** Pediculosis, *Pediculus humanus capitis*, Pediatrics, Head lice, Dimethicone, Infestation, School, Children

## Abstract

**Background:**

Head lice most commonly affect children, ages 3 to 11. Concerns exist about the safety and efficacy of pesticide-based treatments. Published studies suggest dimethicone is a potentially safe and effective non-toxic treatment, but have not evaluated 100 % dimethicone in a pediatric population. The objectives were to evaluate the efficacy and safety of 100 % dimethicone for the treatment of head lice in children, monitored by school nurses.

**Methods:**

This was a multi-site, open-label study of a 100 % dimethicone gel for the treatment of head lice in a pediatric population. Children (ages 3–12) suspected of infestation with head lice were evaluated by school nurses at six schools and daycare programs in New York and New Jersey. Inclusion criteria were presence of at least three live lice, or one live louse and 10 viable eggs (eggs found within 1.27 cm of the scalp) and no use of any head lice treatment within four weeks of enrollment. Counts of live lice and viable eggs found in 58 subjects were tracked at baseline (Day 0) and on Day 1, Day 7, and Day 14 after treatment.

**Results:**

After 1 day of treatment with 100 % dimethicone, 98.30 % of subjects were free of live lice and 55.20 % were free of viable eggs. On day 14, 96.50 % were still free of live lice, and 80.70 % were free of viable eggs. All subjects were monitored by the school nurse at baseline and throughout the study period for adverse effects, including scalp erythema, excoriation, flaking and edema. There was one adverse event of skin irritation lasting 10 min, and no serious adverse events reported. Overall, scalp conditions improved from the baseline: 10 subjects (17.5 %) reported mild to moderate scalp erythema on day 1, compared with only one subject (1.7 %) on day 14; 8 subjects (14.3 %) reported mild scalp excoriation on day 1, with none reporting on day 14.

**Conclusions:**

100 % dimethicone was found to be a safe and highly effective treatment for pediatric head lice. Because dimethicone avoids pesticide exposure and resistance issues, dimethicone should be considered as a first-line treatment for head lice.

**Trial Registration:**

NCT02213055 Date of registration: August 8, 2014

**Standards of reporting:**

The CONSORT 2010 Checklist was consulted during the review of this manuscript. Please note that sections pertaining specifically to randomized controlled trials (RCT’s) were not applicable.

## Background

Head lice, or *Pediculus humanus capitis*, cause an estimated 6 to 12 million infestations per year in the U.S., most commonly affecting children 3 to 11 years of age [1]. Head lice affect all socio-economic groups. Infestations spread regardless of the cleanliness of a home or school environment, or of personal hygiene. It is a significant public health issue due to the high anxiety for children and parents associated with infestation, as well as missed school days. Despite the American Academy of Pediatrics’ recommendation not to restrict infested children from school, there are districts that continue to have a no-nit policy and guardians who keep children out of school [2].

The most common treatments are pesticide-based, over-the-counter remedies containing permethrin (1 %) or other pyrethroid insecticides. Prescription-only options include high-dose permethrin (5 %), malathion (0.5 %), benzyl alcohol lotion (5 %), ivermectin lotion (0.5 %) and spinosad suspension (0.9 %) [3].

Pesticide-based treatments are coming under increasing scrutiny regarding safety and efficacy. Specifically, exposures to neurotoxic pesticides have been linked to lowered IQ, diminished attention span, other neurodevelopmental issues and childhood cancers [4–6]. Additionally, studies have found insecticide resistance in head lice, [7–12] particularly to permethrin and malathion. Given these concerns, an effective and safe alternative pesticide-free treatment is desirable.

Dimethicone (also spelled dimeticone) is a silicone-based polymer that works mechanically to lubricate hair to aid the removal of nits and lice, while physically occluding the respiratory system of the louse. Burgess found that dimethicone potentially eliminates the pesticide-resistance issue as “the blockage [of dimethicone coating the louse] inhibits water excretion, which causes physiological stress that leads to death either through prolonged immobilisation or, in some cases, disruption of internal organs such as the gut” [13].

Several published trials have established plausibility for the use of dimethicone in pediatric lice treatment, demonstrating it may be more effective than conventionally-recommended pesticide-based pediculocides [13–26]. These studies include concentrations of 4-96 % dimethicone in vivo. Only one published trial evaluated the in vitro ovicidal efficacy of 100 % dimethicone. No previously published study has examined the in vivo safety and efficacy of a 100 % dimethicone-based treatment for head lice. Dimethicone-based treatments are commonly used in the European Union and even recommended as a first-line treatment in some Australian jurisdictions [27]. However, they are not a first line treatment in the U.S. and are not listed as one of the recommended treatments in the American Academy of Pediatrics’ (AAP) 2012 Red Book® [28]. Dimethicone is listed in the AAP's 2015 Clinical Report as an alternative treatment [29].

The objectives of this study were to evaluate the efficacy and safety of 100 % dimethicone gel for the topical treatment of head lice in a pediatric, school-centered population.

## Methods

This was a multi-site, open-label study of a 100 % dimethicone gel [LiceMD, manufactured by Combe, Inc, subsequently renamed as LiceMD Pesticide Free] for the treatment of head lice in children. The 100 % dimethicone liquid has a viscosity of 350 centistokes and is composed of a single viscosity polymer.

Children (ages 3–12) suspected of infestation with head lice were evaluated from May 2009 to June 2013 by school nurses at six schools and daycare programs in New York and New Jersey who were trained in all study procedures. Subjects were referred to the school nurse for head lice evaluation by school staff members, parents/guardians, or were diagnosed as part of routine school head checks. Written informed consent for participation in the study was obtained from the subject’s parent or guardian. Additionally, each child nine years of age or older was asked to read and sign an assent form if he or she agreed to participate in the study.

Children could be included as subjects if they met the following criteria as determined by the school nurses, who were trained in how to detect lice using a standard visual census technique: presence of at least three live lice or one live louse and 10 viable eggs (eggs found within 1.27 cm of the scalp). The subject could not have used any head lice treatment within four weeks of enrollment, as reported to the nurse by the parent/guardian. A child could be enrolled more than once in the study only if he or she had not used any other lice treatment or home remedy within the past four weeks. Four subjects were each re-enrolled once in the study. Children were excluded if they had no hair, extremely short hair, a chronic scalp disorder, or were currently taking an antibiotic. Nurses were instructed to ask the parent/guardian about antibiotic use.

Once children were enrolled, guardians were given a copy of the product use directions, the test product, and a lice removal comb. The guardian treated the child at home with the product according to the manufacturer’s instructions. Treatment procedures were as follows: First, apply the product to dry hair, then wait 10 min. Next, with product still in the hair, separate hair into small sections and comb hair to remove lice and eggs. LiceMD® products contain a patented comb specially designed to remove lice and their eggs from hair [30]. Finally, shampoo hair thoroughly with regular shampoo and warm water. After treating the child, the guardian completed the “First Application” Parent Diary and Observation Form in the treatment log.

The first school day following the initial application of dimethicone, the school nurse examined the subject, checking for live lice and viable eggs while visually inspecting the condition of the child’s scalp. If any live lice were found (or if the child had nits and the child’s school had a “no nits” policy), the guardian was notified and advised to immediately apply a second dimethicone treatment. If a second application was done, the guardian was asked to complete the “Second Application” Parent Diary and Observation Form. If any scalp condition score was higher (worse) than the baseline score, the child was checked daily on school days until the irritation resolved. If a third application was needed, the same procedure was followed, and the guardian was asked to complete the “Third Application” page of the treatment log.

The guardian was asked to re-inspect the child’s scalp and hair each day for lice, eggs and nits for 13 days. If lice or viable eggs were seen, the parent was asked to notify the school nurse. The school nurse confirmed the presence and instructed the parent to treat.

On all subjects, seven days (±2 days) and fourteen days (±2 days) after the initial application of treatment, the school nurse conducted a head check for lice and eggs and completed an Egg/Lice/Scalp Condition Form. If the nurse noticed any live lice, the guardian was notified and asked to do another treatment.

Fourteen days (±2 days) after the first treatment, the school nurse conducted another head check on the treated child and completed another Egg/Lice/Scalp Condition Form. If there was still a live lice infestation, the school nurse notified the guardian and discussed further treatment options. Guardians then completed the fourteen day product use questionnaire.

To comply with best practices in ethical approval and consent, this research study was conducted in accordance with the Declaration of Helsinki and was approved prior to the commencement by the HackensackUMC Institutional Review Board (HIRB protocol number Pro00000685). Informed consent was obtained from each participant’s parent or guardian prior to enrollment. The signed consent document included a statement informing parents/guardians that their child’s identity will remain confidential if the results of the trial are published.

The protocol and clinical dataset will be made available upon request to any scientist wishing to use them for non-commercial purposes.

For the statistical analysis, the live lice and viable egg data were analyzed separately, using logistic regression to model the presence or absence of lice/eggs as a function of time, defined by days of treatment, and the other subject characteristics. The correlation between lice/egg counts for each subject on different days was accounted for by using a Generalized Estimating Equation (GEE) approach.

## Results

### Demographics

**Fig. 1:**
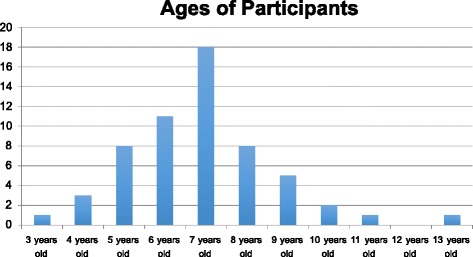
Subject age

A total of 58 subjects were included in the study. Subject characteristics included age, gender, hair amount (thickness), hair length and number of applications. The subjects were aged between 3.5 and 13 years old, with a mean of nearly 7 years (6.98) and a standard deviation of 1.73, as summarized in Fig. 1. Although study eligibility called for subjects ages 3–12, one 13 year old student was inadvertently enrolled. Statistical analysis showed that age did not affect the rate of decrease in the presence of viable eggs due to treatment, i.e. there was no difference in the effectiveness of the treatment with age.

Definitions for hair amount and hair length were not supplied on the questionnaire and were determined by the school nurse and/or the parent filling out the form with the nurse’s assistance. The inclusion of these characteristics was mainly to explore the possibility of varying product effectiveness with these characteristics. No significant differences in effectiveness were found with respect to gender, age or hair characteristics. Table 1 displays summary statistics of the data.

**Table 1:** Subject characteristics

Variable	Values	Number of subjects
Gender	Female	45
	Male	13
Hair amount	Thin	14
	Medium	23
	Thick	20
Hair length	Short	15
	Long	38
	Very long	5
Final Number of treatment applications	1	43
	2	10
	3	5

We enrolled 97 subjects from six schools, resulting in 58 evaluable charts. The 39 subjects not evaluable were excluded due to reasons detailed in Table 2, including issues with documentation (incomplete or missing study forms) or not meeting inclusion criteria.

**Table 2:** Subject exclusions

Reason	Number of participants
Protocol was not Followed at Enrollment	5
Protocol was not Followed during Study	1
Documentation Issue at Enrollment	1
Documentation Issue during Study	10
Did not have Live Lice	8
Had another Treatment during the Study	3
Did not meet Inclusion Criteria	6
Did not Return to School	5

The study included a comparison group in which those opting to use a treatment other than LiceMD could enroll and still be followed for outcomes according to the same criteria in the experimental arm. All subjects chose to enroll in the experimental arm of the study.

### Efficacy

**Fig. 2:**
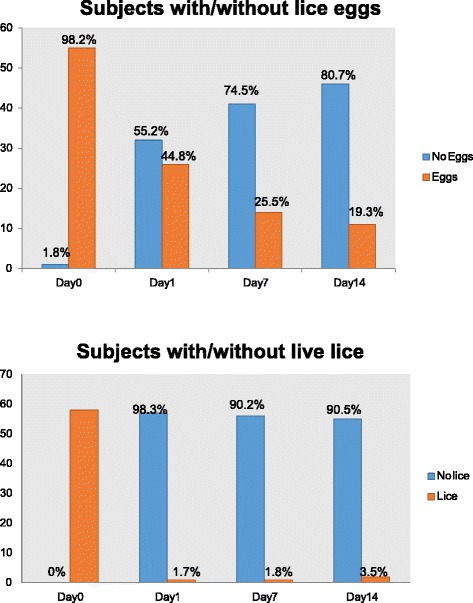
Efficacy of 100 % dimethicone treatment

The data consist of counts of live lice and viable eggs found in 58 subjects at baseline (Day 0) and on Day 1, Day 7, and Day 14 after treatment as shown in Fig. 2. After one day of treatment, 98.30 % of subjects (57 of 58) were free of live lice and 55.20 % (32 of 58) were free of viable eggs. At diagnosis, 55 subjects had viable eggs with three subjects meeting enrollment criteria for three or more live lice. On day 14 of the study, 96.50 % of subjects (55 of 57) were still free of live lice, and 80.70 % (46 of 57) were free of viable eggs. If a child was still found with live lice on day 14, the school nurse notified the guardian to discuss further treatment options, as subjects could not immediately re-enroll in the study. The removal of eggs was facilitated by the viscosity of the product and the lice comb provided, which together eased removal.

Of the 58 subjects, 43 received a total of one treatment, ten received two treatments and five received three treatments [Table 1]. Subjects received a second or third treatment if lice or viable eggs were confirmed by the school nurse.

### Safety

All subjects were monitored by the school nurse at baseline and throughout the study period for adverse effects, including scalp erythema, excoriation, flaking and edema. One adverse event was reported by a parent/guardian during the study, which was transcribed by the school nurse on the Adverse Event Report form as “Irritation on cheek at time of shampoo application” which occurred “10 min after shampoo was washed off.” The nurse also reported that the irritation lasted 10 min and that no medical attention was needed. There were no reports of significant adverse events during the study. Overall, scalp conditions as assessed by the school nurse improved during the two week study period: 10 subjects (17.5 %) reported mild to moderate scalp erythema on day 1, compared with only one subject (1.7 %) on day 14; 8 subjects (14.3 %) reported mild scalp excoriation on day 1, with none reporting on day 14.

## Discussion

This study demonstrates the efficacy and short-term safety for the topical use of 100 % dimethicone for treating pediatric pediculosis. While prior studies have shown efficacy for dimethicone-containing products, to our knowledge this is the first study to document the in vivo safety and efficacy for 100 % dimethicone. Additionally, this study design utilized school and daycare settings to evaluate children for head lice, which is of practical importance as many children with lice are identified and followed by school nurses. There were several limitations for this study: this was an open label single arm design with no comparison group. Additionally, this study had a short term assessment for adverse events.

### Limitations

Originally enrollment was estimated to be 200 subjects, as determined by a questionnaire distributed to the school nurses, asking the approximate number of head lice cases per school year. The study team then estimated the number of expected cases presenting at each participating school during the anticipated enrollment period from May 2009 through June 2011. This enrollment period was later extended to enroll additional subjects, because only six schools out of 19 actively enrolled subjects and the number of subjects enrolled at each school was less than anticipated. School nurses encountered multiple challenges including limitations with communicating instructions and follow-up with the parents/guardians. The nurses also encountered time restrictions due to high student volume in some schools, which at times proved challenging to complete the necessary amount of study paperwork. The study necessitated many volunteer hours from participating school nurses. Efforts to keep school nurses engaged in the study and reminded of protocol procedures included regular e-newsletters and updates, phone calls and in-person visits by the study coordinator and research nurse to assess needs and answer questions.

Study enrollment concluded in June 2013. Funding restrictions limiting enrollment time occurred in December 2010 when the sponsor was purchased by another company and discontinued funding this research. At that time, The Deirdre Imus Environmental Health Center® at Hackensack University Medical Center continued needed funding until the study’s conclusion.

## Conclusions

A 100 % dimethicone product is a safe and highly effective head lice treatment for children and may serve as a potentially less toxic and less resistance prone alternative to pesticide-containing products. Given its safety and efficacy record, dimethicone should be considered as a first-line treatment for pediatric head lice.
