# Erratum to: Safety and efficacy of a 100 % dimethicone pediculocide in school-age children

**DOI:** 10.1186/s12887-016-0547-4

**Published:** 2016-01-21

**Authors:** Erin Speiser Ihde, Jeffrey R. Boscamp, Ji Meng Loh, Lawrence Rosen

**Affiliations:** The Deirdre Imus Environmental Health Center®, Hackensack University Medical Center, 30 Prospect Ave, Hackensack, NJ 07601 USA; Hackensack University Medical Center, The Joseph M. Sanzari Children’s Hospital, 30 Prospect Avenue, Hackensack, NJ 07601 USA; Department of Mathematical Sciences, NJ Institute of Technology - University Heights, Newark, NJ 07102 USA

Following the publication of the original article [1], an error was noticed by the authors. The statistics shown in Figure Two do not accurately reflect the results outlined in the text. To correctly reflect the text, the “subjects with/without live lice” graph in Fig. 2 (Fig. 1 here) should state 98.2 % for day 7 and 96.5 % for day 14 (at current the graph states 90.2 % for day 7 and 90.5 % for day 14). The correct figure is attached to this erratum, for your reference.

**Fig. 2:**
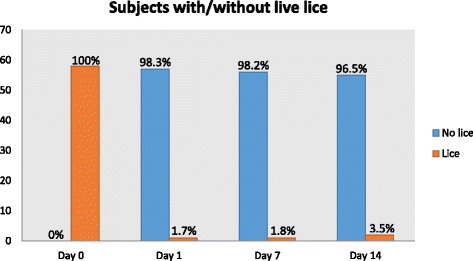
ᅟ
